# Systemic Lupus Erythematosus and Periodontal Disease: A Complex Clinical and Biological Interplay

**DOI:** 10.3390/jcm10091957

**Published:** 2021-05-02

**Authors:** Bouchra Sojod, Cibele Pidorodeski Nagano, Glenda Melissa Garcia Lopez, Antoine Zalcberg, Sophie Myriam Dridi, Fani Anagnostou

**Affiliations:** 1 Service d’Odontologie, Hôpital Universitaire Pitié Salpêtrière (AP-HP), 75013 Paris, France; bouchrasojod@gmail.com (B.S.); m.e.l.i.843@gmail.com (G.M.G.L.); pbca@hotmail.fr (A.Z.); 2 Faculté de Chirurgie Dentaire-Garancière, Université de Paris, 75006 Paris, France; 3 B3OA, CNRS UMR 7052-INSERM U1271, Université de Paris, 75010 Paris, France; cibele.nagano@gmail.com; 4 Faculté de Chirurgie Dentaire, Université Côte d’Azur, 06300 Nice, France; dr.sm.dridi@free.fr

**Keywords:** systematic lupus erythematosus, periodontal disease, risk factors, autoimmune and inflammatory diseases, periodontitis, periopathogens

## Abstract

Reports on the association of periodontal disease (PD) with systemic lupus erythematosus (SLE) have regularly been published. PD is a set of chronic inflammatory conditions linked to a dysbiotic microbial biofilm, which affects the periodontal tissues, resulting eventually in their destruction and contributing to systemic inflammation. SLE is a multi-system chronic inflammatory autoimmune disease that has a wide range of clinical presentations, touching multiple organ systems. Many epidemiological studies have investigated the two-way relationship between PD and SLE, though their results are heterogeneous. SLE and PD are multifactorial conditions and many biological-based hypotheses suggest common physiopathological pathways between the two diseases, including genetics, microbiology, immunity, and environmental common risk factors. By focusing on recent clinical and translational research, this review aimed to discuss and give an overview of the relationship of SLE with PD, as well as looking at the similarities in the immune-pathological aspects and the possible mechanisms connecting the development and progression of both diseases.

## 1. Introduction

Systematic lupus erythematosus (SLE) is an autoimmune disease, which is characterized by the loss of self-tolerance and immune complex-mediated inflammation, and can affect almost every system in the body, with varying degrees of severity [1,2]. The clinical course of the disease is described by recurrent acute or chronic inflammation episodes, leading to the dysfunction of several organs, for example, kidneys, joints, and the skin. In this respect, the oral cavity is not spared. Patients with SLE may present with some oral manifestations, including a wide spectrum of oral mucosal ulceration, such as cheilitis, erythematous patches, honeycomb plaques, discoid lesions, lichen planus (LP)-like lesions, hyposalivation, and xerostomia [3,4]. SLE patients also exhibit a high prevalence of dental caries [5] and an increased number of missing teeth. The aforementioned clinical manifestations result in a negative impact of patients’ oral condition on their quality of life [6]. Moreover, several recent literature have reported that patients with SLE present also a higher risk of periodontitis [7], suggesting a potential association between the two conditions.

Periodontal diseases (PD) comprising gingivitis and periodontitis are highly prevalent diseases worldwide. The prevalence of gingivitis was reported to range from 38% to 85% [8], while severe forms of periodontitis affect ~11% of the global adult population [9]. Gingivitis, the mildest form of periodontal disease, is caused by bacterial biofilms deposited on dental surfaces, subsequent to inadequate self-performed oral hygiene procedures. Gingivitis is characterized by a reversible inflammatory response confined in the gingiva. Its onset and progression may be modified by local factors and/or systemic conditions [8]. Periodontitis, on the other hand, a multifactorial chronic disease caused by polymicrobial synergy of dysbiotic biofilms in susceptible hosts [10], is characterized by the inflammatory destruction of the periodontium, resulting in the irreversible loss of the supporting tooth apparatus, including alveolar bone and, eventually, tooth loss [11]. The effects of periodontitis are not constrained to the oral cavity. Numerous epidemiological, interventional and experimental studies show that periodontitis is associated with several non-communicable pathologies [12], as well as with an increased risk of mortality [13]. It is associated with cardiovascular disease, stroke [14], diabetes [15], adverse pregnancy outcomes [16], pulmonary disease [17]. In addition to these pathologies, periodontitis has been shown recently to be also associated with several other diseases, including metabolic disease and obesity, Alzheimer’s disease [18], certain cancers [12], inflammatory bowel disease [19], and immunoinflammatory diseases, such as rheumatoid arthritis [20,21,22]. Growing evidence suggests that oral dysbiosis, as well as pathogens associated with periodontitis could be involved in the pathophysiology of autoimmune inflammatory diseases, including the SLE.

As aforementioned, the complex multifactorial disease of SLE, characterized by an excessive autoimmune response in the body, represents a major diagnostic and therapeutic clinical challenge [23]. This disease is of unknown etiology and genetic and environmental factors contribute to its susceptibility [24]. Studies on SLE patients (and on mouse models of lupus) have implied the contribution of almost every cell type of the immune system in either the induction or amplification of the autoimmune response, as along with the promotion of an inflammatory environment that exacerbates tissue damage (arthritis, glomerulonephritis, etc.) [25]. Pathogenic autoantibodies mediate the cell damage, which is directed against nucleic acids and protein complexes [1]. Infection is regarded as a trigger for autoimmune diseases and is responsible for controlling the SLE systemic activity. Periodontitis, and specifically oral dysbiosis, could be a contributing factor in sustaining the inflammatory response observed in SLE.

Considering the aforementioned, it was hypothesized that there is an interplay between PD and SLE; the environmental and genetic factors involved in SLE may also contribute to PD pathogenesis while PD may critically act in the initiation and/or the maintenance of the immune-inflammatory response that occurs in SLE. The objective of this article was, therefore, to identify and discuss the evidence regarding the two-way relationship between PD and SLE. The present review was conducted through the PubMed/MEDLINE database, searching for articles written in English and published from 1990 to 2020. The keywords were searched in MeSh (Medical Subject Headings), and the terms used to target peer-reviewed articles were: (systematic lupus erythematosus) AND ((periodontitis) OR (periodontal disease)).

## 2. Epidemiological Evidence for the Association between PD Parameters and SLE

### 2.1. The Impact of SLE Activity on PD

The first literature article dealing with the association between PD and SLE published in 1981 was a case report presenting a 17-year-old female who exhibited edematous gingiva and spontaneous bleeding, and thus was diagnosed with SLE and megakaryocytic thrombocytopenia [26]. Another publication reported the case of an 18-year-old female with SLE who presented severe periodontal involvement manifested by generalized gingival recession [27]. In this case, lack of predisposing aspects for chronic periodontitis was interpreted by the authors in favor of direct association between SLE and the periodontal status. A case of acute necrotizing ulcerative gingivitis (ANUG) was also reported in a patient with SLE in 1985 [28]. A higher incidence of gingivitis was also observed in juvenile SLE patients in contrast to that observed in healthy children and adolescents [29].

The prevalence and severity of PD, specifically periodontitis, in the SLE patients have been the subject of several studies (summarized in Table 1). The most studied clinical parameters of PD were pocket depth, bleeding on probing, gingival recession, and clinical attachment loss, which is the representative of cumulative periodontal destruction. According to literature reports, the prevalence of periodontitis in SLE patients varies between 60% and 94% [30]. Other studies have also reported the increased prevalence of PD in the SLE patients compared to the healthy controls [31,32,33,34,35]. In agreement with these studies, compared to adult population, higher prevalence (almost 70%) was observed in the SLE patients.

However, data regarding the severity of clinical parameters of periodontitis in the SLE patients compared to either healthy volunteers, or patients with PD without SLE are conflicting. Recent controlled studies have reported greater severity of PD in the patients with SLE, exhibiting more clinical attachment loss [34], and/or increased pocket depth [36]. Moreover, reduced periodontal probing depths in the SLE patients (compared to the control group) was noticed [32,37,38,39,40]. Severity of the periodontal parameters monitored was similar in the SLE patients and the control subjects; however, chronic periodontitis occurred earlier in the SLE patients [30]. Absence of a statistically significant difference between the results from controls and SLE cases could be ascribed to the use of various anti-inflammatory drugs. This could also raise the question around the impact of the immunosuppressive treatment of SLE on the PD parameters. Moreover, in a meta-analysis, it was shown that the overall risk of periodontitis was significantly increased by 1.76 (95% CI 1.29–2.41, *p* = 0.0004) in the patients with SLE, compared to the respective controls. However, there was no statistically significant difference in individual parameters of periodontitis such as probing depth (*p* = 0.06) and clinical attachment loss (*p* = 0.08) between the SLE cases and healthy cases [7]. Likewise, a recent meta-analysis involving 80,633 subjects showed a significant increase in the prevalence of periodontitis (odds ratio = 5.32, 95% CI 1.69–16.78, *p* = 0.004), while no significant difference was observed in the incidence of severe periodontitis between the patients with SLE and healthy controls [41]. In the SLE patients, a higher prevalence of bleeding on probing, higher mean clinical attachment loss, and similar values of mean pocket depth, gingival index, and plaque index were observed [41]. These conflicting findings might be attributed to many factors, including (i) differences in the definition and the clinical measurement of PD; (ii) differences in the scoring of SLE activity and damage; (iii) the presence of potential confounding factors of comorbidity (e.g., smoking and stress); (iv) the use of anti-inflammatory drugs for the treatment of SLE; and (v) differences in the genetic and environmental backgrounds of the studied populations; as well as (vi) the clinical study design (e.g., sample size, type of study, etc.).

### 2.2. Impact of PD on the Pathogenesis of SLE

Recent clinical evidence has demonstrated the implication of PD in the pathogenesis of SLE. A nationwide, population-based, retrospective case-control study explained the association between the history of PD and newly diagnosed SLE (OR, 1.21; 95% CI, 1.14–1.28; *p* < 0.001), which was both dose- and time-dependent [42]. Given that smoking is a common risk factor for both PD and SLE, the aforementioned association is weak and attributed to the lack of information on the individual smoking status of patients. Nevertheless, in another recent randomized clinical trial, the influence of periodontitis treatment on the manifestation of SLE was investigated. The authors reported that PD treatment improved response to immunosuppressive therapy, suggesting that PD may be a modifiable risk factor for SLE [31]. The strength of this association is weak due to the cross-sectional nature of this study, the limited information collected on the progression of SLE, and the use of immunosuppressive treatment. On the other hand, Wang et al. [34] reported that patients with periodontitis had 26.94 times higher risk of having SLE than the patients without periodontitis; these results highlighted the role of periodontal interventions in the prevention and risk assessment of SLE. Moreover, Bae and Lee [43], in a recent study, analyzed the associations from genome-wide association studies on European population, using PD as an exposure and SLE as an outcome with Mendelian randomization. Interestingly, they found a weak but significant evidence that periodontitis is causally related with an elevated risk of SLE incidence in line with the published epidemiological studies.

## 3. Biological Basics for a Potential Relationship between PD and SLE

In addition to epidemiological data, several studies, presented in the following section, have reported that shared genetic and environmental risk factors, as well as the activation of immune pathways underlying local pathological outcomes for the two conditions have led to the relationship between SLE and PD.

### 3.1. Genetic Link

Several loci and genetic variants have been identified to be associated with SLE [2]; however, only three studies have addressed the link of SLE with PD. Notably, Kobayashi et al. [32,39] assessed the distribution of the FcγPIIA, FcγPIIIA and FcγPIIIb genotypes and alleles in 71 Japanese SLE patients with and without PD, and in healthy subjects with and without PD, and reported that the PD–SLE connection might be associated to the polymorphism of Fcγ receptor. Specifically, these authors reported that FcγRIIA-R131 and the combination of FcγRIIA-R131 and FcγRIIB-232T alleles are strongly in association with SLE and periodontitis. Japanese SLE patients with the combined FcγR risk alleles experienced more severe periodontal tissue destruction than other SLE patients. Further studies are needed, however, to confirm the association of FcγR with SLE in other ethnic populations. Schaefer et al. [47] sought to elucidate the shared genetic basis of either SLE or rheumatoid arthritis (RA) with aggressive periodontitis. These authors not only identified PRDM1 and IRF5 as candidate genes that play a role in IFN-signaling and have genome-wide association with SLE, but they reported that the extent of shared risk loci is limited. Further studies are required to determine the pathogenic genetic link between the two diseases and related polymorphism(s).

Besides genetics, epigenetic modifications as a result of the interaction of bacterial metabolites and epigenetic enzymatic reactions, are also involved in pathogenesis of both PD and SLE [48]. Epigenetic mechanisms, such as DNA methylation and histone modification can trigger breakdown of immunological homeostasis of periodontal environment, but further investigations are needed to a more complete understanding of cyclic inflammation/dysbiosis process.

### 3.2. Potential Mechanisms Linking SLE with the PD Pathogenesis

Potential mechanisms linking the SLE with the pathogenesis of PD include (i) the effects of systemic immune dysregulation on subgingival microbiota; (ii) the SLE-induced imbalance between pro-inflammatory and anti-inflammatory cytokines, which seems to be the cause of tissue damage [36]; (iii) the activation of autoreactive B cells and dysregulation of several other immune cell types, including macrophages, neutrophils, CD4+ T cells, and dendritic cells [48].

#### 3.2.1. Effect of SLE-Associated Systemic Immune Dysregulation on Subgingival Microbiota

It has been reported that systemic inflammatory disorders such as diabetes, RA and inflammatory Bowel disease, might contribute to the destruction of periodontal tissue by disrupting the balance between host and oral microbiota [19,49]. A recent study examined the effects of SLE on the subgingival microbiota and reported that the SLE patients had a dysbiotic subgingival microbiota with higher subgingival bacterial load, and experienced major alterations in bacterial composition with a shift to greater quantities of pathogenic bacteria, including *Prevotella oulorum, P. Oris, P.nigrescens, S. noxia, Lachnospiraceae*, and *Leptotrichia*, even in periodontally healthy sites [36]. In another study, *Candida albicans* and *Lactobacilli* were observed in higher proportions in the SLE patients and *A. actinomycetemcomitans* in juvenile SLE patients as compared with controls [50]. SLE disease activity and severity has been correlated to changes of the PD-associated microbiota [51]. *Treponema denticola* and *Tannerella forsythia* were also in increased quantities in SLE-active periodontal sites in comparison to that of SLE-inactive and healthy controls [52]. SLE-associated inflammatory changes in the periodontium providing a source of nutriments by altering the subgingival microbiota may favor bacterial interactions that lead to the increased susceptibility to PD [53]. The dysbiotic microbial community may play, therefore, a central role in the mechanistic link between the two conditions, though further studies are warranted to define it.

#### 3.2.2. SLE-Induced Imbalance Between Pro-inflammatory and Anti-Inflammatory Cytokines

In patients affected by both PD and chronic inflammation-driven disorders, changes in the oral microbiota have been linked to the increased local inflammation [19,53]. For instance, modified patterns of cytokines expression in gingival tissue and of cytokines levels in CGF and in saliva have been reported in inflammatory bowel disease [19], diabetes [53] and RA [54]. Indeed, in SLE, the salivary concentrations of IL-6 and IL-17A were significantly higher in the SLE/PD patients compared to the controls/PD subjects. Moreover, IL-6, IL-17A, and IL-33 levels were increased in the SLE/PD patients compared to the SLE patients without PD [36,55,56]. Increased levels of the same cytokines have been observed also in the saliva of RA patients [54]. Additionally, increased levels of IFN-γ, IL-10, IL-1β, and IL-4 were observed in the saliva of patients with SLE, even in the absence of PD [55]. Specifically, IL-17 involved in both PD and SLE pathogenesis was reported to play a key role in the process of inflammation/dysbiosis [57]. In CGF of SLE/PD patients’ levels of visfatin, an adipokine involved in pro-inflammatory response, are increased [58]. Furthermore, the altered levels of IL-1β, IL-8, G-CSF, IFN-γ, and CMP-1 in gingival crevicular fluid (CGF) have been associated to worsened periodontal conditions in patients with juvenile SLE [50]. Some of these cytokines specifically IFN-γ, IL-10, and IL-4 were also increased in the serum samples of the SLE patients and were linked to the disease manifestation [1]. Pessoa et al. reported serum cytokine dysregulation in SLE patients to be dependent on SLE activity [51]. However, the involvement of cytokines in the periodontal tissue destruction is difficult to interpret due to the various effects of the same cytokines at different stages of SLE, as well as on the pathogenesis of PD. Furthermore, systemic use of the anti-inflammatory drugs (e.g., corticoids) affects the local production of some cytokines (e.g., IL-1β and IL-18) in the gingiva [56] and, therefore, may modify the local inflammatory response to the dysbiotic flora and the periodontium destruction.

### 3.3. B-Cell Hyperactivity

Destructive lesions are dominated by B-lymphocytes and plasma cells [11], and interestingly, in SLE, B-cell hyperactivity was proposed as the basic mechanism for the generation of autoantibodies [2]. The interaction between antigen presenting cells, abnormally activated T cells, and hyperactive B cells results in the production of soluble autoantibodies of the IgG isotype, as well as various cytokines. The secreted autoantibodies form immune complexes by binding autoantigens and, in turn, complement fixation, or engaging Fcγ receptors on several different cell types, leading to tissue damage [32,39]. If, and how, the increased autoantibodies in SLE contribute to the chronic tissue damage resulting from periodontitis remains to be elucidated.

## 4. Potential Mechanisms Underlying the Links between PD and the Pathogenesis of SLE

PD and oral dysbiosis are implicated in several autoimmune diseases, including SLE, and several pathogenic mechanisms have been proposed to explain this association [59]. In the pathogenesis of SLE, infections may play a pivotal role in addition to the genetic, hormonal, and environmental aspects [2,60]. SLE patients secrete large amounts of antibodies against various oral bacteria. Specifically, serum antibody titers against PD-associated bacteria such as *A. actinomycetemcomitans, P. gingivalis, T. denticola*, and *C. ochracea* were higher in the patients who were positive for anti-dsDNA antibodies significantly correlated with anti-dsDNA titers and reduced levels of complement [61]. Moreover, antibodies to *A. actinomycetemcomitans* were associated with higher disease activity.

Infectious agents can trigger autoimmune diseases through various mechanisms, including molecular mimicry, epitope spreading, alterations in self-antigens, and immune cell activation in genetically susceptible individuals [62]. More precisely, it has been reported that periopathogens might cause the excessive activation of immune response in the SLE by retaining a high expression of TLRs in periodontal tissues, and in turn, leading to the acceleration of the onset and progression of autoimmune reactions [55]. Indeed, the expression levels of TLR-2 TLR-4 are increased in both PD [63] and SLE [64]. These findings suggest that periopathogens (e.g., *P. gingivalis*) stimulate the expression of TLR-2 and TLR-4 in the periodontium and activate the mechanisms of local and systemic autoimmunity related to the SLE, which might be, at least partially, associated with the disease in the SLE patients.

Besides these exogenous pathogen-associated molecular patterns, TLRs can bind with damage-associated molecular patterns (DAMPs) released by damaged tissues or “endogenous” apoptotic cells [64]. DAMPs can induce inflammation and immune response in the absence of infection, promote maturation/activation of various immune cells, as well as production of pro-inflammatory cytokines, and break tolerance to self-antigens contributing to autoimmune diseases like SLE. In fact, the concentration of HMGB1 (a well-known DAMP) in gingival crevicular fluid (GCF), as well as the number of HMGB1-positive cells are higher in the inflamed gingival epithelium of patients with periodontitis than that of healthy subjects [65]. HMGB1 expression was also enhanced in the SLE patients and was correlated with the index of SLE disease activity [66].

The chronically inflamed periodontium is the site where immune tolerance to citrullinated epitopes is broken, and the production of ACPAs commences even before the clinical symptoms of RA by many years [21]. Occurrence of ANCA-positive sera in the SLE patients with periodontitis is high (83.3%) compared to that in the SLE patients without periodontitis [33]. SLE is characterized by the production of autoantibodies (e.g., antinuclear, anti-dsDNA, anti-Sm, anti-Ro, anti-CL, and anti-β2GPI antibodies) coupled with patient failure to suppress them. Some of these antibodies cause disease because of their antigen specificity. *P. gingivalis* (a keystone periodontal pathogen) is suspected to contribute to other disease-specific autoantibody responses [21]. Interestingly, patients with active SLE who harbored *P. gingivalis* alone or in combination with *T. denticola* significantly exhibited higher intraoral anti-CL and anti-β2GPI antibodies than the patients without these bacteria. Moreover, anti-CL and anti-β2GPI antibody levels were correlated with periodontal attachment loss, increased C-reactive protein level, and erythrocyte sedimentation rate [34].

## 5. Conclusions

Overall, to date, despite the controversial results, the available data clearly suggest a possible bidirectional association between PD and SLE that should be considered in the management of SLE patients. Prospective clinical studies that enroll large numbers of very well-defined patients (in terms of both PD and SLE) and respective control subjects, are needed to confirm the causal association and to elucidate the biochemical and immunological interaction between the two diseases.

## Figures and Tables

**Table 1:** Epidemiologic observational studies investigating the association between PD and SLE.

Reference	Design	Quality Rating	Demographics	Periodontal Assessment Methodology	Results
Rutter-Locher et al. 2017 (England) [7]	Systematic review and meta-analysis	Good	487 SLE patients 896 controls	PI BOP PD CAL Residual teeth	Statistically significant increased risk of periodontitis in patients with SLE compared to controls.
Voger et al. 1981 (USA) [26]	Case report	Poor	A 17-year old black female	BOP, PD	The patient was diagnosed with a generalized severe gingivitis associated with SLE and amegakaryocytic thrombocytopenia.
Nagler et al. 1999 (Israel) [27]	Case report	Poor	An 18-year old female	BOP, PD, Recession	Severe periodontal loss was manifested by gingival recession. Focal lymphoepithelial lesion was found in the gingival subepithelium. Periodontitis was found in the SLE patient.
Jaworski et al. 1985 (Korea) [28]	Case report	Poor	A 35-year old Korean female	Gingival aspect Pain Swelling Adenopathy	The patient presented ANUG. SLE and the therapeutic amounts of steroids may have contributed to the increased severity of the oral disease.
Fernandes et al. 2007 (Brazil) [29]	Case control study	Fair	48 children and adolescents with SLE, 48 children and adolescents as controls	PI GI	Patients with SLE presented poor oral hygiene. Higher incidence of gingivitis
Fabbri et al. 2014 (Brazil) [31]	Randomized controlled trial	Fair	32 SLE/ periodontitis-treated patients 17 SLE/ periodontitis-untreated patients	PD CAL GBI	Prevalence of periodontitis among SLE patients initially selected for the study was 89%. PD treatment improved response to immunosuppressive therapy in SLE patients.
Kobayashi et al. 2007 (Japan) [32]	Case control study	Fair	46 SLE/periodontitis patients 25 SLE patients 58 periodontitis patients 44 controls	Number of missing teeth PD CAL BOP PI	64.8% of SLE patients had periodontitis. The combination of stimulatory FcγRIIa and inhibitory FcγRIIb genotypes was associated with the risk of periodontitis in SLE patients in the Japanese population.
Novo et al. 1999 (Venezuela) [33]	Comparative study	Fair	30 patients with SLE 30 patients with RA 20 controls	PD BOP Bone loss (estimated in a periapical Rx)	60% of SLE patients had periodontitis. An association was found between ANCA and periodontitis in SLE patients.
Wang et al. 2015 (Japan) [34]	Case-control study	Fair	53 SLE patients 56 controls	PD CAL Pg and Td levels in gingival sulcus Levels of serum anti-CL and anti-β2GPI antibodies	Prevalence of periodontitis among SLE patients was 79%. Periodontitis was associated with an increased production of anti-β2GPI-dependent anti-CL antibodies in patients with SLE.
De Pablo et al. 2015 (England) [35]	Case-control study	Fair	105 SLE patients 484 controls	PD	Periodontal disease was more common among individuals with SLE.
Correa et al. 2017 (Brazil) [36]	Case-control study	Fair	52 SLE patients 52 controls	PD CAL BOP PI Dental personal hygiene Subgingival bacterial composition	Prevalence of periodontitis among SLE patients was 53%.
Zhang et al. 2017 (Chine) [37]	Case-control study	Fair	108 SLE patients 108 controls	PI BOP GI Bone loss PD CAL	Chinese SLE patients were likely to suffer from higher odds of PD.
Figueredo et al. 2008 (Brazil) [38]	Case-control study	Fair	16 JSLE patients 14 controls	PD PI GBI CAL Expression of Il-18, IL-1β, elastase activity in GCF	Higher levels of active elastase in GCF from inflamed sites in JSLE patients. Greater risk of tissue degradation and periodontal attachment loss of JSLE patients compared to healthy juvenile controls.
Kobayashi et al. 2003 (Japan) [39]	Case control study	Fair	42 SLE/periodontitis patients 18 SLE patients 42 periodontitis patients 42 controls	Number of missing teeth PD CAL BOP, PI	70% of SLE patients had periodontitis. FcγRIIa -R131 allele was associated with the risk of periodontitis in SLE patients in the Japanese population.
Mutlu et al. 1993 (England) [40]	Case-control study	Fair	27 SLE patients 25 controls	PD	Patients with SLE significantly had lower periodontal probing depths compared to healthy controls.
Wu et al. 2017 (Japan) [42]	Retrospective Case-control study	Fair	7204 SLE patients 72040 controls	Number of periodontitis-related visits History of PD	A higher risk of SLE was significantly associated with a history of PD. Prevalence of periodontitis among SLE patients was 35%.
Al Mutairi et al. 2015 (Kingdom of Saudi Arabia) [44]	Case-control study	Fair	25 SLE patients 50 controls	PI BOP CAL PD Residual teeth	Periodontal health was not different between SLE patients and controls.
Rhodus and Johnson 1990 (the USA) [45]	Case series	Poor	16 females with SLE	PD CAL	93.8% of studied patients presented periodontitis.

**Note:** BOP: bleeding on probing, PD: probing depth, SLE: systemic lupus erythematosus, ANUG: acute necrotizing ulcerative gingivitis, PI: plaque index, GI: gingival index, CAL: clinical attachment loss, RA: rheumatoid arthritis, ANCA: antineutrophil cytoplasmic antibodies, GBI: gingival bleeding index, GCF: gingival crevicular fluid, JSLE: juvenile systemic lupus erythematosus. The quality rating of the epidemiological studies performed according to Oxford center for evidence-based medicine 2011. OCEBM Levels of Evidence. (Electronic resource). URL: https://www.cebm.ox.ac.uk/resources/levels-of-evidence/ocebm-levels-of-evidence (accessed on 27 April 2021). “Poor” corresponds to the level 4, “fair” corresponds to levels 2 and 3, and “good” corresponds to level 1 [46].
